# Is it me? Verbal self-monitoring neural network and clinical insight in schizophrenia

**DOI:** 10.1016/j.pscychresns.2015.10.007

**Published:** 2015-12-30

**Authors:** Adegboyega Sapara, Dominic H. ffytche, Michael A. Cooke, Steven C.R. Williams, Veena Kumari

**Affiliations:** a Department of Psychology, Institute of Psychiatry, Psychology and Neuroscience, King’s College London, London, UK; b Department of Old Age Psychiatry and Department of Neuroimaging Sciences, Institute of Psychiatry, Psychology and Neuroscience, King’s College London, London, UK; c Department of Neuroimaging Sciences, Institute of Psychiatry, Psychology and Neuroscience, King’s College London, London, UK; d NIHR Biomedical Research Centre for Mental Health, South London and Maudsley NHS Foundation Trust, London, UK

**Keywords:** Psychosis, fMRI, Self-awareness, Anterior cingulate, Medial frontal gyrus, Putamen, Birchwood insight scale

## Abstract

Self-monitoring, defined as the ability to distinguish between self-generated stimuli from other-generated ones, is known to be impaired in schizophrenia. This impairment has been theorised as the basis for many of the core psychotic symptoms, in particular, poor clinical insight. This study aimed to investigate verbal self-monitoring related neural substrates of preserved and poor clinical insight in schizophrenia. It involved 40 stable schizophrenia outpatients, 20 with preserved and 20 with poor insight, and 20 healthy participants. All participants underwent functional magnetic resonance imaging with brain coverage covering key areas in the self-monitoring network during a verbal self-monitoring task. Healthy participants showed higher performance accuracy and greater thalamic activity than both preserved and poor insight patient groups. Preserved insight patients showed higher activity in the putamen extending into the caudate, insula and inferior frontal gyrus, compared to poor insight patients, and in the anterior cingulate and medial frontal gyrus, compared to healthy participants. Poor insight patients did not show greater activity in any brain area compared to preserved insight patients or healthy participants. Future studies may pursue therapeutic avenues, such as meta-cognitive therapies to promote self-monitoring or targeted stimulation of relevant brain areas, as means of enhancing insight in schizophrenia.

## Highlights

• We investigate neural substrates of both preserved and poor clinical insight in schizophrenia.
• We report an association between neural networks involved in verbal self-monitoring and clinical insight.
• We present evidence of functional deficits in the putamen and inferior frontal gyrus associated with poor insight.

## 1 Introduction

One important indicator of clinical outcomes in psychotic disorders is the level of insight a patient has into his/her mental condition (Drake et al., 2007). Poor clinical insight has been closely associated with poor medication compliance, more frequent relapses and hospital admissions, poor long-term outcomes and, overall poor global functioning (Amador and David, 2004). Patients with poor insight, characteristically demonstrate a lack of awareness of the presence of a mental disorder, an inability to identify their psychotic experiences as being abnormal (misattribution of symptoms) and/or failure to recognise or identify the need for treatment (David, 1990). As there are limited data on the underlying cause or explanation of this phenomenon, there are few clinical strategies specifically aimed at enhancing insight of affected patients (Shad et al., 2007).

The ability to accurately self-appraise and monitor self-related information may be crucial to having a good insight in psychosis (Kircher and David, 2003, Shad et al., 2007). Kircher and Leube (2003) postulated that intact self-awareness is dependent on intact self-monitoring processes, and that a subconscious inability to label self-generated impulses as originating from “self” underpins core psychotic symptoms such as somatic passivity and thought disorders. In many studies, patients with schizophrenia are found to show poor monitoring of self-generated stimuli in the visual, tactile and verbal domains and misattribute them to other sources (Raveendaran and Kumari, 2007). Shad et al. (2007), in their model of the neurobiology of poor clinical insight in schizophrenia, theorised that impaired self-awareness results in misattribution of symptoms, as awareness of symptoms is crucial in them being correctly attributed to those of the disorder (i.e. good clinical insight). They further linked aberrant functioning of the neural substrates implicated in poor self-appraisal and monitoring to misattribution of symptoms, and thus to poor insight, in schizophrenia (Shad et al., 2007).

A number of recent studies (Gerretsen et al., 2014, Shad and Keshavan, 2015, van der Meer et al., 2013) have focussed on the neurobiology of insight in schizophrenia using paradigms that directly or indirectly involve monitoring of the self or self-relevant information. Associations have been found between poor clinical insight and increased connectivity in the self-referential network with the left insula during rest (Gerretsen et al., 2014), better clinical insight and activation of the inferior frontal gyrus, anterior insula and inferior parietal lobule during self-reflection (van der Meer et al., 2013); and between symptom unawareness and activation of many areas, including the prefrontal, parietal and limbic areas, with more specific associations between symptom misattribution and localised regions within the prefrontal cortex and basal ganglia, during a self-awareness task (Shad and Keshavan, 2015).

This present study aimed to investigate the neurobiology of clinical insight in psychosis further by examining functional alterations within the verbal self-monitoring neural network in patients with poor as well as preserved clinical insight, relative to each other and a group of healthy participants. The functional neuroanatomy of verbal self-monitoring in healthy people includes the anterior cingulate cortex (ACC), dorsolateral prefrontal cortex (DLPFC), left inferior frontal cortex, putamen, temporal cortex, posterior cingulate and the inferior parietal cortex (Allen et al., 2005, Kumari et al., 2010b, Raveendaran and Kumari, 2007, Shergill et al., 2001). Functioning of many of these areas, based on recent studies of insight in psychosis (Gerretsen et al., 2014, Sapara et al., 2014; Shad and Keshavan, 2015; van der Meer et al., 2013), appears to be involved in maintaining a good insight in schizophrenia. Previous studies have consistently shown reduced superior-middle temporal lobe activity during variants of the verbal self-monitoring task (Fu et al., 2006, Shergill et al., 2000a, Kumari et al., 2010b) but no published study, to our knowledge, has examined verbal self-monitoring performance or the functioning of the associated neural network in relation to the level of insight in schizophrenia.

Based on the existing models implicating self-monitoring deficits in poor insight in psychosis (Shad et al., 2007) and recent fMRI findings (Gerretsen et al., 2014, Sapara et al., 2014; Shad and Keshavan, 2015; van der Meer et al., 2013), we hypothesised that patients with poor insight, compared to those with preserved insight, will show less accurate self-monitoring performance and aberrant fMRI response in the verbal self-monitoring neural network. Poor, but not preserved insight, patients were expected to show markedly impaired performance and performance-related fMRI activations relative to healthy participants.

## 2 Methods

### 2.1 Participants and design

The study involved 60 right-handed participants in total. The sample included 40 people with a Diagnostic and Statistical Manual of Mental Disorders (DSM, fourth edition DSM-IV, APA, 1994) diagnosis of schizophrenia (Structured Clinical Interview for DSM-IV, SCID; First et al., 1995). Of these, 20 patients were pre-selected to have preserved insight and 20 to have poor insight out of a larger pool of 70 patients (see Creation of low and high insight groups). All included patients were required to be on stable doses of antipsychotic medication for at least three months and in the stable (chronic) phase of the illness. Of 40 patients initially included, 14 patients (7 poor and 7 preserved insight) had to be excluded: four patients (2/group) had movement artefacts (i.e. rotations >5° or translations >5 mm), four poor insight patients failed to follow the task instructions, and performance data from 1 poor and 5 preserved insight patients were unavailable (equipment failure) during fMRI. Twenty healthy participants, screened to exclude neuropsychiatric conditions using the SCID (non-patient version (SCID-NP) and matched, on average to the two patient groups, for age and sex, were studied for comparison purposes, with 16 providing useable data (*n*=3, movement artefacts; *n*=1 technical failure). Of those remaining in the final sample, 19 patients (9 poor insight, 10 preserved insight) and 11 healthy participants were also included in our earlier study (Sapara et al., 2014).

The study procedures were approved by the research ethics committee of the Institute of Psychiatry and South London and Maudsley NHS Trust, London. All participants provided written informed consent.

### 2.2 Clinical assessment

Insight was assessed using the Birchwood insight scale (BIS) (Birchwood et al., 1994). The BIS assesses three dimensions of clinical insight (David, 1990), namely (i) the presence of a mental illness (items 2 and 7), (ii) the need for treatment (items 3,4,5 and 6), and (iii) the identification of symptoms as abnormal (items 1 and 8). Each BIS item is rated as ‘agree’, ‘disagree’ or ‘unsure’, giving an item score of 1 for unsure, and 0 or 2 for agree and disagree, depending on whether agreement with the statement indicates good insight (items counterbalanced for response valence). Item 4 “My stay in hospital is necessary” was omitted, as we did not include any inpatients. This yielded a maximum score of 14 (from the remaining 7 items) in this data set instead of 16 observed on the full BIS. For classification of insight levels, Birchwood (1994) suggested a score of 9 (out of 16) as the minimum for good clinical insight. In addition, in all patients symptoms were assessed using the Positive and Negative Syndrome scale (PANSS) (Kay et al., 1987) and predicted IQ was assessed in all participants using the National Adult Reading Test (NART) (Nelson and Willison, 1991).

### 2.3 Creation of preserved and poor insight groups

We classified patients into “preserved” or “poor” insight groups, rather conservatively by defining preserved insight as a score of 13 or above and poor insight as 8 or less (out of a maximum 14) to ensure distinct insight levels in preserved and poor insight groups (Sapara et al., 2014). Patients were supervised while completing the BIS. The BIS has adequate internal consistency and satisfactory test–retest reliability (Birchwood et al., 1994), and BIS insight scores correlate positively with scores on clinician-rated measures of insight such as the Scale to Assess Unawareness of Mental Disorders (SUMD; Amador and David, 2004) and the Expanded Schedule of Assessment of Insight (SAI-E; David and Kemp, 1997) (Young et al., 2003; Sapara et al., 2007).

### 2.4 fMRI paradigms and procedure

All participants performed a self-monitoring task (Kumari et al., 2010b) whilst undergoing fMRI. Participants were presented with single words on a computer screen (visible for 750 ms, inter-stimulus interval 16.25 s), viewed (wearing fMRI compatible glasses where needed) via a prismatic mirror fitted in the radiofrequency head coil, as they laid in the scanner, and were instructed to read each word aloud. The participant’s speech was transformed in real time through a software programme and a DSP.FX digital effects processor (Power Technology, California, USA), amplified by a computer sound card, and relayed back through an acoustic MRI sound system and pneumatic tubes within the ear protectors at a volume of 91 dB (SD±2 dB). The volume of the feedback was sufficient to overcome the bone conduction of the participant’s own voice.

The verbal feedback was either: (a) own voice (Self); (b) own voice lowered in pitch by 4 semitones (Self-distorted); (c) voice of another person matched on participant’s sex (Other); or (d) another person’s voice with the pitch lowered by 4 semitones (Other-distorted). Participants registered their responses regarding the origin of feedback by using the button box with the ‘self’ button press for their voice, the ‘other’ button press for ‘other’ voice or the ‘unsure’ button if they were unsure about the nature of the feedback. Words ‘self’, ‘other’ and ‘unsure’ were displayed on the screen and outlined in black after each response. Accuracy of the responses was recorded online. Participants’ occasional failures to press a button were recorded as non-responses. In total, 64 words (e.g. begin, dressed, quick, smooth, living, restful etc.) were presented, 16 in each condition (Self, Self-distorted, Other and Other-distorted) occurring in a pseudo-random order. The entire experiment lasted about 16 min. Participants were familiarised, prior to fMRI session, with the experimental procedures until they understood what they were required to do while undergoing fMRI.

### 2.5 Image acquisition

Echoplanar MR brain images were acquired using a 1.5 T GE Signa system (General Electric, Milwaukee WI, USA). A quadrature birdcage head coil was used for RF transmission and reception. In each of 12 near axial non-contiguous planes (slice thickness=7.0 mm, interslice gap= 0.7 mm) parallel to the inter-commissural plane, T2*−weighted MR images depicting blood-oxygenation-level-dependent (BOLD) contrast was acquired. This resulted in partial brain coverage excluding slices at the vertex but covering key regions of interest based on the apriori hypothesis including DLPFC, ACC and other regions involved in self-monitoring. A clustered acquisition sequence was used, with 1.1 s of scanner noise (TE=40 ms, 70° flip angle), and a relative silent period of 2.15 s for each stimulus within a TR of 3.25 s and the inter-stimulus interval of 16.25 s, yielding five brain volumes for each trial. A clustered acquisition sequence was used to minimise artefacts associated with overt speech during image acquisition (see Amaro et al. (2002) for full technical details).

### 2.6 Statistical analyses

#### 2.6.1 Demographic, clinical and behavioural measures

The groups were compared on age, education and (NART) IQ, using analysis of variance (ANOVA), followed by independent sample *t*-tests. Preserved and poor insight groups were compared on age of illness onset (defined as age at first appearance of psychotic symptoms, as reported retrospectively by patients and confirmed by documented record where possible), duration of illness, PANSS symptoms and medication using independent sample *t*-tests.

Group differences in task performance [the percentage of correct, incorrect or unsure responses and reaction time (RT) to correct responses] were examined (separately) by Group (low insight, high insight, healthy participants)×Source (Self, Other)×Distortion (undistorted voice, distorted voice) ANOVA with Group as a between-subjects factor and Source and Distortion as within-subjects factors, followed by lower order ANOVAs and post-hoc mean comparisons as appropriate. Effect sizes for group differences were estimated as *partial eta*^2^.

All analyses were carried out using SPSS 22. Alpha level for significance testing was *p*=0.05, 2-tailed, unless otherwise stated.

#### 2.6.2 Functional MRI

##### 2.6.2.1 Pre-processing

For each participant, the volume functional time series were motion corrected (*x*, *y* and *z* translation, pitch, roll, yaw), transformed into stereotactic space using the EPI template in SPM (affine transformation *x*, *y* and *z*, 16 nonlinear iterations, 7×9×7 basis functions), spatially smoothed with a 10 mm FWHM Gaussian filter and band pass filtered using statistical parametric mapping software (SPM5; http://www.fil.ion.ucl.ac.uk/spm5).

##### 2.6.2.2 Models and inferences

All analyses were run on brain activity during trials with correct answers. There were too few errors for the Self condition in most healthy participants and some patients to allow a meaningful analysis of brain responses during errors. fMRI data were analysed using a two-stage random effect procedure (Friston et al., 1999) using statistical parametric mapping software (SPM8; http://www.fil.ion.ucl.ac.uk/spm8). The first stage identified subject-specific activations. We then identified performance-related neural activations (height threshold *p*<0.001; family wise error (FWE) corrected for multiple comparisons at the cluster level *p*) using one-sample *t*-tests separately in the three groups. The second stage involved separate ANOVAs within SPM8 for each task condition to identify regions of activity (height threshold *p*<0.05; FWE-corrected at the cluster level *p*<0.05) differentiating two or more groups (healthy participants vs poor/preserved insight, poor insight vs preserved insight) during the Self and Other conditions. Group differences in brain activity during distorted conditions were not analysed because of (i) a much reduced accuracy during distorted conditions (thus reduced power), and (ii) overlapping activation patterns during the four conditions in healthy participants (Supplementary materials, Appendix 1 and Figure 2). Some group differences that were present only at the uncorrected level, but occurred within the self-monitoring network found in previous studies with this task (Kumari et al., 2010b), are also reported (Table 2).

Next, the subject-specific activation contrast image values were extracted for the regions (peak voxel) differentiating the two patient groups from each other, and from healthy participants. These values were examined (within SPSS and SPM 8) for their possible relationships with performance (% correct) first using ANOVA with brain activity as the dependent variable and relevant Groups as the between-subjects variable, and then, in order to understand the contribution of (varying) number of trials that provided fMRI data to differences in brain activity of the three study groups, using analysis of co-variance (ANCOVA) with brain activity as the dependent variable, relevant Groups as the between-subjects variable, and performance (accuracy and RT for relevant task condition) and NART IQ as the covariates.

## 3 Results

### 3.1 Demographic, clinical and behavioural measures

The three study groups were comparable in age [*F*(2,39)=2.27, *p*=0.12]. There was a significant Group effect in education [*F*(2,39)=3.81, *p*=0.03]; healthy participants had more years of education than the preserved insight [*t*(27)=2.29, *p*=0.03] and poor insight patients [*t*(27)=2.29, *p*=0.03]. There was a significant Group effect in NART IQ [*F*(2,39)=3.15, *p*=0.05]; healthy participants and preserved insight patients were comparable but healthy participants had higher NART IQ than poor insight patients [*t*(27)=2.57, *p*=0.02] (Table 1).

The preserved and poor insight groups were comparable in age at illness onset, duration of illness and symptoms, and were prescribed similar doses of antipsychotic medication (all *p*>0.11) (Table 1). By design, the groups differed significantly in the level of insight [total BIS score, *t*(24)=11.29, *p*<0.001].

### 3.2 Performance

For percent of correct responses (Table 1), there were main effects of Source [*F*(1,39)=6.99, *p*=0.01, *partial eta*^2^=0.15; indicating higher accuracy during Self than Other conditions]; Distortion [*F*(1,39)=48.89, *p*<0.001, *partial eta*^2^=0.56; indicating higher accuracy during undistorted than distorted conditions] and Group [*F*(2,39)=3.83, *p*=0.03, *partial eta*^2^=0.16]. Follow-up analysis of Group effect showed that healthy participants had higher accuracy than both preserved [*F*(1,27)=4.66, *p*=0.04, *partial eta*^2^=0.15] and poor insight patients [*F*(1,27)=5.99, *p*=0.02, *partial eta*^2^=0.18]. Although, as expected, preserved insight patients had numerically better performance than poor insight patients, this difference was not statistically significant [*F*(1,24)=0.96, *p*=0.34]. There was no Source×Distortion or Source×Distortion×Group interaction (all *p* values >0.19).

For percent of incorrect responses, there were main effects of Source [*F*(1,39)=6.95, *p*=0.01, *partial eta*^2^=0.15], indicating fewer errors during Self (mean=11.61%, SD=13.39) than Other (22.17%, 18.11) conditions, and of Distortion [*F*(1,39)=37.04, *p*<0.001, *partial eta*^2^=0.49] indicating fewer errors during undistorted (10.04%, 10.13) than distorted (23.73%, 13.94) conditions. All other effects were non-significant (all *p* values >0.20). Similarly, for percent of unsure responses, there were main effects of Source [*F*(1,39)=6.37, *p*=0.02, *partial eta*^2^=0.14] indicating fewer unsure responses during Self (6.47%, 11.26) than Other (9.97%, 10.85) conditions, and of Distortion [*F*(1,39)=9.09, *p*<0.001, *partial eta*^2^=0.19] indicating fewer unsure responses during undistorted (4.39%, 7.31) than distorted (12.05%, 16.71) conditions. There was also a trend for the main effect of Group [*F*(2,39)=2.59, *p*=0.09, *partial eta*^2^=0.12], with poor insight patients (13.34%, 14.21) showing more unsure responses than healthy participants (5.66%, 8.00) and preserved insight patients (6.25%, 5.41). All other effects were non-significant (all *p* values >0.59].

For RTs to correct responses (Table 1), there were main effects of Source [*F*(1,39)=21.09, *p<*0.01, *partial eta*^2^=0.351], indicating faster RTs during Self than Other conditions, and of Distortion [*F*(1,39)=70.03, *p*<0.001, *partial eta*^2^=0.64] indicating faster RTs during undistorted than distorted conditions. There was also a significant main effect of Group [*F*(2,39)=7.75, *p*=0.001, *partial eta*^2^=0.28] with slower RTs in both preserved [*F*(1,27)=14.09, *p*=0.001, *partial eta*^2^=0.34] and poor insight patients [*F*(1,27)=11.57, *p*=0.002, *partial eta*^2^=0.30] relative to healthy participants. All other effects were non-significant (all *p* values >0.10).

### 3.3 fMRI

#### 3.3.1 Generic performance-related activations

Performance-related activations in all study groups are noted in Appendix 1 (Supplementary materials). Healthy participants activated a large neural network involving the thalamus, superior-middle temporal gyrus, inferior frontal gyrus and inferior parietal lobe with successful monitoring of own or someone else's voice in both distorted and undistorted conditions, with a remarkable overlap across the four conditions (Figure 2, Supplementary materials). The parahippocampal gyrus, posterior and medial frontal gyrus areas were significantly deactivated during one or more conditions. The preserved insight group showed significant activation and de-activation of many of the same areas that were present in the healthy group. The poor insight group showed activation in fewer and relatively smaller clusters and did not show significant de-activation of any brain area during the Self or Self-distorted condition.

#### 3.3.2 Group differences in performance-related brain activations

Group differences in performance-related activations are presented in Table 2, and described below.

##### 3.3.2.1 Healthy participants vs poor insight patients

Healthy participants showed greater activity than poor insight patients in the thalami (bilaterally) and the lentiform nucleus (left), during the Self condition; and in a large cluster including the posterior cingulate, inferior parietal lobule, insula, superior temporal gyrus (all left sided) and the thalamus (right) during the Other condition. Poor insight patients did not show greater activity in any area during any task condition.

##### 3.3.2.2 Healthy participants vs preserved insight patients

**Fig. 1:**
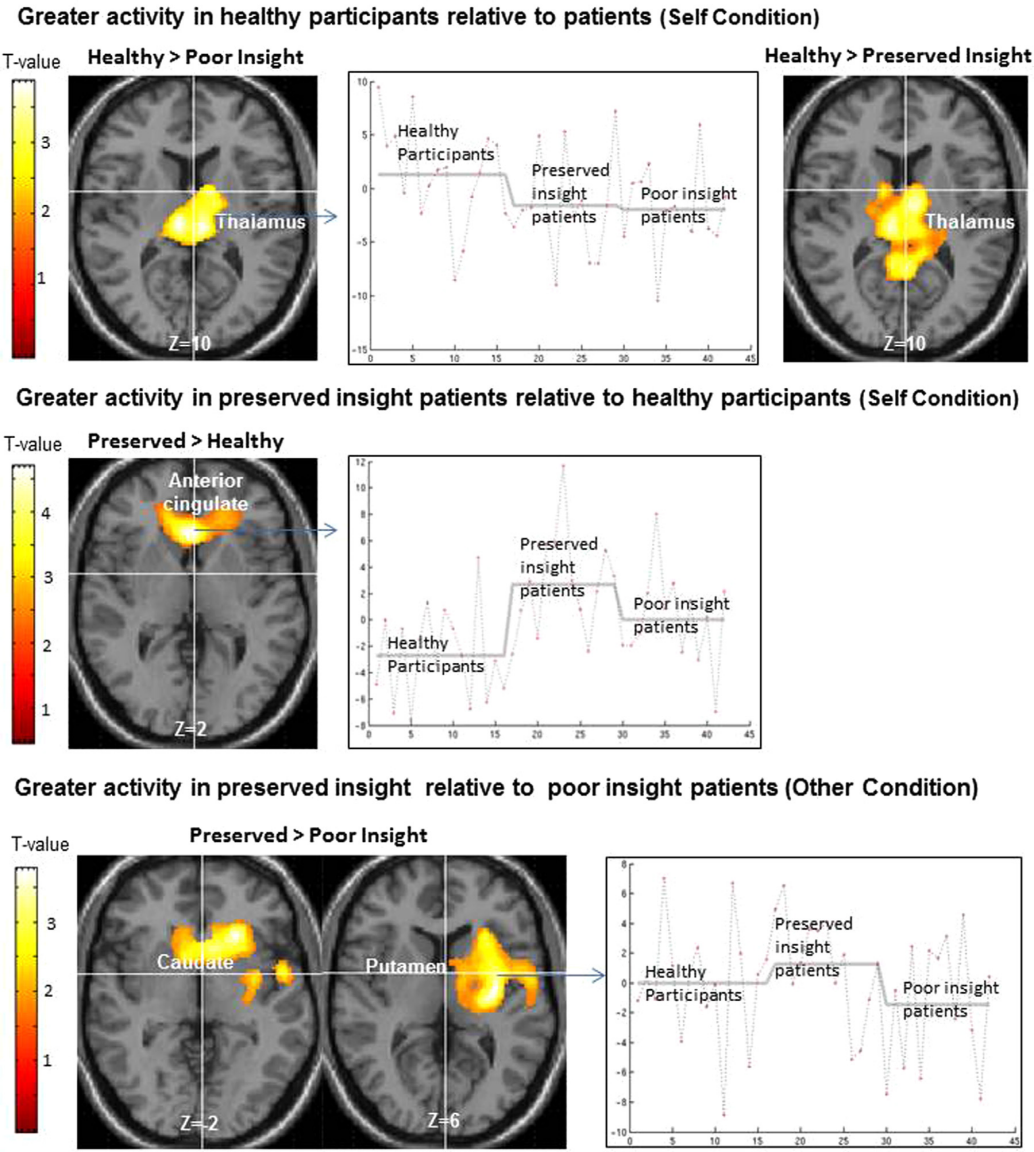
Axial sections of a standard MNI brain with areas of greater activity in the preserved insight group, compared to the healthy participant and poor insight groups superimposed (height threshold *p*<0.05, cluster-corrected *p*<0.05). The MNI *Z* co-ordinate is given below each image. Right of image=left hemisphere.

Healthy participants, relative to preserved insight patients, again showed greater activity in the thalami (bilaterally) and the right posterior cingulate (Self condition). Preserved insight patients showed greater activity in the ACC and medial-middle frontal gyrus (Self condition) (Fig. 1).

##### 3.3.2.3 Preserved insight vs poor insight patients

Preserved insight patients showed greater activity (Other condition) in the putamen extending into the caudate, insula and inferior frontal gyrus (Fig. 1). Poor insight patients did not show greater activity in any area during any task condition.

#### 3.3.3 Group differences in brain activations after co-varying for performance and NART IQ

The strength of the group differences described above was not affected by co-varying for accuracy, indicating the differences were unrelated to the ‘number of correct trials’ which varied across groups and might have influenced statistical power to detect activations in each group (Table 3). Group differences in activations also remained unaffected after co-varying for RT and NART IQ (Table 3).

## 4 Discussion

The present study aimed at investigating the association between preserved and poor clinical insight and fMRI activity within the self-monitoring neural network in stable schizophrenia outpatients. It tested the hypothesis that patients with poor insight, compared to those with preserved insight, will show less accurate verbal self-monitoring and aberrant fMRI response in the verbal self-monitoring neural network. Furthermore, it expected to find markedly impaired performance and performance-related fMRI activations in poor, but not preserved insight, patients relative to healthy participants. Behaviourally, healthy participants showed the best (and significantly better than both patient groups) and poor insight patients showed the worst performance of all groups. Concerning group differences in brain activity (Table 2), poor insight patients, relative to preserved insight patients, showed lower activity (Other condition) in the putamen extending into the caudate, insula and inferior frontal gyrus (Fig. 1). They also showed significantly less activation of the thalamus (Self and Other conditions) and of the posterior cingulate, inferior parietal lobules, superior temporal gyrus regions (Other) relative to healthy participants. Preserved insight patients did not differ from healthy participants during the Other condition but they too showed lower thalamic and posterior cingulate activity and, in addition, increased ACC and medial frontal activity (relative to healthy participants) during the Self condition. Broadly, the observed effects at the behavioural (though not formally significant) and neural levels (with the exception of increased ACC and medial frontal activity in the preserved insight group relative to healthy participants) are in line with our a priori hypotheses.

The behavioural data (better performance during the Self than during the Other, and during Undistorted than during Distorted conditions) as well as brain activation patterns (Supplementary materials, Appendix 1 and Figure 2) in healthy participants were consistent with those found in previous studies (Fu et al., 2008, Fu et al., 2006, Kumari et al., 2010b). The thalamic deficit observed in both patient groups of this study is also consistent with a previous study (Kumari et al., 2010b) which showed a thalamic activation deficit (peak co-ordinates −8, −30, 14) during the same task in patients with schizophrenia regardless of their symptoms or task performance. Aberrant thalamic structure and functions have also been reported in other schizophrenia studies (Andreasen et al., 1994, Bak et al., 2014, Kumari et al., 2007).

Insight related activation differences between poor and preserved insight patients (lower activity in the poor insight group) were present in the putamen and caudate, extending into the insula and inferior frontal gyrus (Table 2, Fig. 1). All of these areas are known to be involved in self-monitoring, serving in the appraisal and attribution of self-generated stimuli (Kumari et al., 2010b, McGuire et al., 1996a, Shergill et al., 2001). Interestingly, the difference between poor and preserved insight patients was observed only while monitoring someone else’s voice as non-self (Other condition). A possible interpretation of this finding might be that correct monitoring of others’ voices also involves the recognition of own voice as distinct from that of others and is comparatively more difficult (thus more power to distinguish the groups) than the Self condition (see performance data). Interestingly, larger putamen size, both at the initial and at the chronic phase of the disorder, has been associated with better illness outcome measured, for example, by lower symptom severity, higher remissions and lower dependence for basic needs (Buchsbaum et al., 2003). Mitelman et al. (2009) further suggested that schizophrenia patients with poor clinical outcomes develop pronounced shrinkage in the putamen during the course of the illness, especially within the first two decades, and this may be a marker of treatment-resistance and poor global outcomes. So far, no study has explored the direct relationship between increased striatal volumes and the preservation of insight (which, as mentioned in the Introduction, also predicts better longer-term clinical outcome), and how these either jointly or independently facilitate good outcomes in schizophrenia. Interestingly, lower inferior frontal activity during self-monitoring has been found to be associated with a poor clinical outcome (less symptom improvement) following cognitive behavioural therapy for psychosis (Kumari et al., 2010a).

Preserved insight patients showed greater brain activity (during Self) in the ACC and medial-middle frontal areas, relative to healthy participants. This difference resulted from both a deactivation in healthy participants and activation of these areas in preserved insight patients (Fig. 1). ACC and medial frontal sub-regions are implicated in a “default” mode of conscious experience (Gusnard et al., 2001). It is possible that preserved insight patients (unlike poor insight patients) were almost as accurate as healthy participants at recognising their own voice correctly (Self condition, healthy participants: mean=92.58%, SD=6.54; preserved insight patients: mean=91.83%, SD=5.34) by maintaining ACC and medial frontal activation, rather than showing a deactivation. Given the possible overlap between the Self condition, which involved processing of own (thus familiar) voice and a relatively low perceptual component, and the default baseline state that itself involves self-awareness, our findings suggest that the default mode of brain action may contribute to preserved insight by supporting self-awareness as theorised by Shad et al. (2007).

The present study is the first to have conducted a head-to-head comparison of neural differences during verbal self-monitoring between preserved and poor insight patients. Since neural differences between these patient groups remained after co-varying for education and NART IQ (Table 3); and the groups were comparable on all other demographic and clinical parameters (Table 1); they are most likely explained by differing self-monitoring ability and insight levels of the two patient groups.

### 4.1 Strengths and limitations of the study

The strengths of the study include the use of distinct (poor and preserved insight) groups of patients with closely matched demographic and clinical characteristics, and a well-established fMRI self-monitoring task, enabling valid comparisons and inferences. Furthermore, known high correlations between the scores on the BIS and clinician-rated measures of insight such as the SAI-E and SUMD (Sapara et al., 2007) allow the present results to be related meaningfully to findings of previously published studies that used clinician-rated measures (Gerretsen et al., 2014, Shad and Keshavan, 2015, van der Meer et al., 2013). However, of the initial 40 patients recruited into the study (20 with preserved insight and 20 with poor insight) and 20 healthy participants, only 26 patients (13 preserved and 13 poor insight) and 16 healthy participants remained in the final sample. The inability of some of the poor insight patients (*n*=4) to comply with the task procedure could possibly be due to their lower cognitive ability (low NART IQ). The final sample, however, still had 13 or more individuals per group. Another limitation is that our participants were predominantly male, so we did not have sufficient statistical power to examine the effects of sex. Higher NART IQ in healthy participants relative to poor insight patients, and higher education in healthy participants relative to both patient groups, may be considered a further limitation although all significant group differences remained significant after co-varying for years of education and NART IQ. Nonetheless, possible group differences in other neurocognitive functions that were not assessed in this study might have contributed to the observed group differences. Lastly, the lack of full brain coverage means that we are not able to exclude the possibility of additional areas outside the self-monitoring network in the superior parietal lobe and vertex differing in activation between groups.

The findings of this study provide direct evidence of the association between neural networks involved in the self-monitoring process and insight in schizophrenia. There was evidence of ACC-medial PFC involvement in maintaining good insight and of putamen and inferior frontal gyrus deficits in poor insight. The findings advance our understanding of the neurological underpinnings of poor insight in psychosis and lend support to the use of possible therapeutic interventions such as meta-cognitive therapies to promote self-reflectiveness (Pijnenborg et al., 2015) or targeted stimulation of relevant brain areas, aimed at promoting insight in schizophrenia.

## Contributors

Adegboyega Sapara, Dominic ffytche and Veena Kumari designed the study. Steven Williams helped with imaging protocol development. Michael Cooke assisted with the data collection. Adegboyega Sapara undertook the statistical analysis and prepared the first draft under Veena Kumari and Dominic ffytche’s supervision. All authors contributed and approved the final manuscript.

## Conflict of interest

The authors declare no conflict of interest.

## Figures and Tables

**Table 1:** Demographics, clinical characteristics and task performance of study groups.

	**Healthy participants**			**Patients**						
**Preserved insight group**				**Poor insight group**		
*n*=16; (13M:3F)			*n*=13; (11M:2F)				*n*=13; (9M:4F)		
**Demographics**	**Mean (SD)**		**Range**	**Mean (SD)**			**Range**	**Mean (SD)**		**Range**

Age (years)	31.81 (9.36)		20–59	31.15 (9.77)			19–52	37.85 (7.43)		26–49
Education (years)	15.81 (2.76)		11–20	13.38 (2.93)			9–20	13.54 (2.30)		11–19
Predicted IQ (NART)^a^	115.67 (8.65)		95–128	110.04 (10.50)			86–122	106.92 (9.66)		90–124
						
**Clinical characteristics**										
bBIS insight score				13.61 (0.62)		13–14		5.97 (1.69)		2–8
Age at illness onset (years)	21.23 (7.06)		12–38		22.69 (5.68)		10–31
Duration of illness (years)	9.92 (7.22)		1–30		15.15 (9.64)		1–24
cPositive symptoms	18.15 (5.10)		10–25		16.23 (4.44)		8–22
cNegative symptoms	18.31 (5.12)		7–27		17.85 (6.84)		8–27
cGeneral psychopathology	35.46 (7.95)		26–56		30.62 (6.96)		21–40
cTotal symptoms	71.92 (15.87)		44–108		64.69 (16.11)		37–86
Antipsychotic dose (chlorpromazine equivalent in mg)	467.08 (400.46)		160–1600		623.80 (392.59)		200–1367
Antipsychotic type				10 patients on atypical, 1 on typical and 2 on both types				7 patients on atypical, 4 on typical and 2 on both types		
			
*Performance	Percentage correct	RT (s)		Percentage correct	RT (s)			Percentage correct	RT (s)	
Mean (SD)	Mean (SD)		Mean (SD)	Mean (SD)			Mean (SD)	Mean (SD)	

Self	92.58 (6.54)	1.66 (0.36)		91.83 (5.34)	2.30 (0.66)			80.29 (19.74)	2.62 (0.88)	
Self-distorted	78.13 (27.58)	2.00 (0.34)		57.69 (35.28)	2.65 (0.55)			63.94 (31.99)	2.55 (0.68)	
Other	85.15 (19.62)	1.96 (0.52)		72.60 (21.12)	2.60 (0.38)			67.31 (25.66)	2.64 (0.69)	
Other-distorted	56.64 (26.95)	2.27 (0.68)		61.54 (31.23)	2.88 (0.66)			51.44 (28.32)	3.02 (0.82)	
Total	78.13 (2.41)	1.97 (0.42)		70.91 (2.22)	2.61 (0.48)			65.75 (4.78)	2.71 (0.73)	

RT= Reaction time for correct responses.

a National Adult Reading Test.

b Birchwood insight scale (BIS).

c PANSS: Positive and Negative Symptom Scale.

**Table 2:** Group differences in performance-related brain activations (height threshold *p*<0.05).

**Groups**	**BA**	**Size**	**Side**	**MNI coordinates**			***T*****value**	**Cluster*****p***
				***X***	***Y***	*Z*	FWE-corrected unless shown in italics
**Healthy participants>poor insight patients**								
**Self**								
Thalamus	n/a	3531	R	8	−20	10	3.38	*0.009*
	n/a		L	−4	−24	12	3.27	
Lentiform nucleus	n/a			−14	2	2	3.21	
Thalamus	n/a			−8	−22	10	3.26	
								
**Other**								
Posterior cingulate	29	6461	L	−2	−44	18	4.59	0.008
Thalamus	n/a		R	2	−20	6	4.21	
Inferior parietal lobule	40		L	−40	−34	26	3.69	
Superior temporal gyrus	41			−46	−26	14	3.58	
Insula	13			−40	−32	18	3.36	
Claustrum	n/a			−32	−12	8	3.33	
								
**Healthy participants>preserved insight patients**								
**Self**								
Thalamus	n/a	5029	L	−8	−6	14	4.04	0.042
	n/a		R	8	−26	10	3.61	
	n/a		L	−4	−18	14	3.40	
Posterior cingulate	29		R	6	−48	12	3.35	
Thalamus	n/a		L	−8	−26	12	3.24	
								
**Preserved insight patients>healthy participants**								
**Self**								
Anterior cingulate cortex	24	2014	L	0	26	2	4.19	*0.040*
Middle frontal gyrus	11			−22	44	−10	3.47	
	11			20	26	−12	2.88	
								
**Preserved insight patients>poor insight patients**								
**Other**								
Putamen (extending into the insula and inferior frontal gyrus)	n/a	4812	L	−30	−14	6	3.77	0.030
Caudate	n/a			−8	18	−2	3.18	
Putamen	n/a			−32	−10	6	3.68	

**Poor insight patients>healthy participants**								
Nil significant in any task condition.								

**Poor insight patients>preserved insight patients**								
Nil significant in any task condition.								

BA=Brodmann Area; MNI=Montreal Neurological Institute.

**Table 3:** ANOVAs and ANCOVAs of group differences in brain activity patterns with performance (accuracy and RT) and NART IQ as covariates.

**Comparison**	**Task condition**	**Brain region**	**Side**	**MNI coordinates**				**ANOVA**		**ANCOVA (with % correct as a covariate)**		**ANCOVA (with RT as a covariate)**		**ANCOVA (with NART IQ as a covariate)**	
***X***	***Y***	***Z***	**BA**	***F*** (1,27)	***p***	***F*** (1,26)	***p***	***F*** (1,26)	***p***	***F*** (1,26)	***p***
**Healthy participants>poor insight patients**	Self	Thalamus	R	8	−20	10	n/a	13.29	0.001	11.49	0.002	15.89	<0.000	15.89	<0.000
Other	Posterior cingulate	L	−2	−44	18	29	21.43	<0.001	20.10	<0.001	24.97	<0.000	24.97	<0.000
**Healthy participants>preserved insight patients**	Self	Thalamus	L	−8	−6	14	n/a	15.27	0.001	15.52	0.001	12.04	0.002	12.04	0.002
**Preserved insight patients>healthy participants**	Self	Anterior cingulate	L	0	26	2	24	17.28	<0.001	16.48	<0.001	13.10	0.001	13.10	0.001
															
								***F*** (1,24)	***p***	***F*** (1,23)	***p***	***F*** (1,23)	***p***	***F*** (1,23)	***p***
**Preserved insight>poor insight patients**	Other	Putamen	L	−30	−14	6	n/a	12.46	0.002	11.68	0.002	12.82	0.002	12.82	0.002

BA=Brodmann Area; MNI=Montreal Neurological Institute; NART= National Adult Reading Test; RT= Reaction time for correct responses.

## Footnotes

Appendix A Supplementary data associated with this article can be found in the online version at doi:10.1016/j.pscychresns.2015.10.007.
