# Supplementary material for: Is it me? Verbal self-monitoring neural network and clinical insight in schizophrenia
Source: Psychiatry Res. 2015 Dec 30;234(3):328–35. doi: 10.1016/j.pscychresns.2015.10.007 (PMC4834462; doi:10.1016/j.pscychresns.2015.10.007)
Supplement: Supplementary file 1 — Supplementary material [file mmc1.doc]

Appendix 1. Individual group brain activations (height threshold *p*<0.001).

| **Healthy participants** | | **Cluster size**  (voxels) | **Side** | **MNI** | | | Voxel  *T* value | Cluster FWE-corrected *p* |
| --- | --- | --- | --- | --- | --- | --- | --- | --- |
| **1. Self** | **BA** |  |  | **X** | **Y** | **Z** |  |  |
| **Increases** | | | | | | | | |
| Superior temporal gyrus | 41 | **17144** | L | -54 | -28 | 10 | 10.47 | <0.001 |
| Superior temporal gyrus | 41 |  | R | 52 | -26 | 0 | 10.13 |  |
| Insula | n/a |  | R | 36 | 10 | 0 | 9.97 |  |
| Inferior frontal gyrus | 9 |  | R | 48 | 2 | 28 | 9.90 |  |
| Superior temporal gyrus | 41 |  | L | -50 | -38 | 22 | 9.87 |  |
| Inferior frontal gyrus | 9 |  | R | 44 | 12 | 22 | 9.61 |  |
| Precentral gyrus | 6 |  | R | 52 | 0 | 34 | 9.57 |  |
| Cingulate gyrus | 31 | **228** | L | -4 | -30 | 30 | 6.63 | 0.05 |
|  |  |  |  | -6 | -30 | 36 | 5.72 |  |
|  |  |  |  | -8 | -30 | 42 | 4.31 |  |
|  |  |  |  | -8 | -36 | 44 | 3.89 |  |
| Precuneus | 7 | **596** | R | 16 | -82 | 34 | 6.49 | 0.001 |
| Cuneus | 18 |  |  | 10 | -80 | 26 | 5.10 |  |
| Precuneus | 7 |  |  | 18 | -78 | 44 | 4.86 |  |
| Cuneus | 18 |  |  | 18 | -70 | 14 | 4.76 |  |
| Cuneus | 18/19 | **806** | L | -18 | -80 | 22 | 5.95 | <0.001 |
|  |  |  |  | -16 | -90 | 40 | 5.80 |  |
|  |  |  |  | -20 | -88 | 36 | 5.66 |  |
|  |  |  |  | -12 | -86 | 24 | 5.65 |  |
| **Decreases** | | | | | | |  | |
| Anterior cingulate gyrus | 11 | **2479** | R | 4 | 42 | -10 | 4.86 | <0.001 |
| Medial frontal gyrus | 11 |  |  | 4 | 48 | -14 | 4.80 |  |
| Middle frontal gyrus | 11 |  |  | -20 | 44 | -14 | 4.55 |  |
|  |  |  |  | -22 | 30 | -14 | 4.48 |  |
|  |  |  |  | -24 | 28 | -18 | 4.42 |  |
| Caudate | n/a | **694** | L | -24 | -40 | 10 | 6.15 | <0.001 |
| Parahippocampal gyrus | 19 |  |  | -30 | -50 | -2 | 5.40 |  |
| **2. Self-distorted** | | | | | | | | |
| **Increases** | | | | | | | | |
| Precentral gyrus | 6 | **7491** | R | 52 | -2 | 28 | 15.26 | <0.001 |
| Superior temporal gyrus | 41 |  |  | 56 | -26 | 8 | 11.62 |  |
| Middle temporal gyrus | 21 |  |  | 58 | -14 | -10 | 11.01 |  |
| Transverse temporal gyrus | 41 |  |  | 48 | -30 | 10 | 10.92 |  |
| Inferior frontal gyrus | 47 |  |  | 44 | 22 | 2 | 10.09 |  |
| Middle frontal gyrus | 46 |  |  | 44 | 16 | 24 | 9.62 |  |
| Superior temporal gyrus | 41 | **6375** | L | -52 | -32 | 12 | 10.64 | <0.001 |
| Precentral gyrus | 6 |  |  | -50 | -8 | 32 | 9.63 |  |
|  |  |  |  | -54 | -4 | 20 | 9.10 |  |
| Transverse temporal gyrus | 41 |  |  | -56 | -24 | 8 | 9.06 |  |
| Precentral gyrus | 4 |  |  | -58 | -4 | 22 | 9.00 |  |
| Precentral gyrus | 6 |  |  | -50 | -10 | 40 | 8.91 |  |
| Thalamus | n/a | **299** | R | 14 | -14 | 6 | 9.98 | <0.001 |
| Thalamus | n/a | **312** | L | -10 | -16 | 6 | 6.58 | <0.001 |
|  |  |  |  | -18 | -26 | 2 | 4.93 |  |
| Cuneus | 18 | **252** | L | -16 | -78 | 30 | 5.93 | <0.001 |
|  |  |  |  | -16 | -82 | 22 | 4.95 |  |
| **Decreases** | | | | | | |  | |
| Caudate | n/a | **910** | R | 8 | 24 | 8 | 9.91 | <0.001 |
|  |  |  | L | -4 | 20 | 6 | 9.50 |  |
|  |  |  |  | -12 | 26 | 4 | 8.63 |  |
| Cingulate gyrus | 24 | **685** | L | -24 | -12 | 38 | 8.56 | <0.001 |
|  |  |  |  | -16 | -8 | 36 | 6.77 |  |
| Caudate | n/a |  |  | -18 | -22 | 28 | 4.87 |  |
| Parahippocampal gyrus | 19 | **1064** | L | -36 | -48 | -2 | 8.11 | <0.001 |
| Caudate | n/a |  |  | -22 | -42 | 12 | 7.59 |  |
| Fusiform gyrus (Temporal) | 20 |  |  | -42 | -28 | -20 | 5.95 |  |
| Cingulate gyrus | 24 | **668** | R | 20 | -10 | 36 | 7.11 | <0.001 |
| Middle temporal gyrus | 37 | **227** | L | -58 | -46 | -12 | 7.55 | <0.001 |
| Caudate | n/a | **838** | R | 26 | -44 | 16 | 7.39 | <0.001 |
|  |  |  |  | 38 | -50 | -8 | 5.66 |  |
| Parahippocampal gyrus | 19 |  |  | 42 | -48 | -8 | 5.61 |  |
| Inferior parietal lobule | 39 | **444** | L | -46 | -66 | 38 | 5.39 | <0.001 |
| Middle temporal gyrus | 39 |  |  | -48 | -64 | 28 | 5.32 |  |
| Inferior parietal lobule | 40 |  |  | -50 | -62 | 38 | 5.24 |  |
| **3. Other** | | | | | | | | |
| **Increases** | | | | | | | | |
| Thalamus | n/a | **1189** | R | 6 | -16 | 6 | 11.58 | <0.001 |
| Superior temporal gyrus | 41 | **6250** | L | -54 | -22 | 8 | 9.54 | <0.001 |
|  |  |  |  | -48 | -32 | 14 | 9.14 |  |
|  |  |  |  | -48 | -28 | 12 | 8.97 |  |
|  |  |  |  | -58 | -20 | 2 | 8.93 |  |
| Insular | 13 |  |  | -38 | 10 | 0 | 8.88 |  |
| Superior temporal gyrus | 22 |  |  | -56 | -44 | 12 | 8.07 |  |
| Superior temporal gyrus | 41 | **3640** | R | 60 | -20 | 8 | 8.65 | <0.001 |
|  |  |  |  | 58 | -22 | 2 | 8.60 |  |
| Inferior parietal lobule | 40 |  |  | 52 | -32 | 22 | 6.87 |  |
| Middle temporal gyrus | 22 |  |  | 50 | -36 | 6 | 6.74 |  |
| Superior temporal gyrus | 42 |  |  | 54 | -34 | 12 | 6.67 |  |
|  |  |  |  | 68 | -30 | 12 | 6.09 |  |
| Precuneus | 7/31 | **906** | R | 12 | -78 | 40 | 7.36 | <0.001 |
|  |  |  |  | 10 | -70 | 24 | 5.93 |  |
| Cuneus | 7 |  |  | 8 | -74 | 30 | 5.62 |  |
| Precuneus | 19 |  | L | -10 | -82 | 40 | 5.01 |  |
| Insular | 13 | **428** | R | 44 | 6 | 4 | 4.32 | <0.001 |
| **Decreases** | | | | | | | | |
| Middle temporal gyrus | 39 | **1236** | L | -28 | -56 | 26 | 9.60 | <0.001 |
| Parahippocampal gyrus | 36 |  |  | -34 | -36 | -14 | 7.33 |  |
| Hippocampus | n/a |  |  | -32 | -46 | 2 | 6.15 |  |
| Middle temporal gyrus | 39 |  |  | -38 | -50 | 4 | 6.08 |  |
| Parahippocampal gyrus | 19 |  |  | -34 | -46 | -2 | 5.99 |  |
| Precentral gyrus | 6 | **943** | R | 32 | -6 | 38 | 7.96 | <0.001 |
| Cingulate gyrus | 24 |  |  | 22 | -10 | 38 | 6.48 |  |
| Cingulate gyrus | 24 | **883** | L | -14 | -4 | 36 | 7.31 | <0.001 |
| Middle frontal gyrus | 8 |  |  | -24 | 16 | 36 | 7.04 |  |
| Hippocampus | n/a | **1197** | R | 32 | -44 | 4 | 6.07 | <0.001 |
| Superior temporal gyrus | 22 |  |  | 38 | -50 | 12 | 6.03 |  |
| Inferior parietal lobule | 40 |  |  | 44 | -56 | 36 | 5.99 |  |
| Hippocampus | n/a |  |  | 32 | -46 | 8 | 5.94 |  |
| **4. Other-distorted** | | | | | | | | |
| **Increases** | | | | | | | | |
| Precentral gyrus | 44 | **6869** | R | 58 | 6 | 14 | 19.50 | <0.001 |
|  |  |  | R | 48 | 4 | 16 | 14.83 |  |
| Superior temporal gyrus | 22/41 |  | R | 58 | 0 | 2 | 11.59 |  |
|  |  |  |  | 56 | -26 | 2 | 8.81 |  |
|  |  |  |  | 62 | -16 | -2 | 8.78 |  |
|  |  |  |  | 56 | -18 | 8 | 7.97 |  |
| Precentral gyrus |  |  | R | 58 | -10 | 34 | 7.67 |  |
| Precentral gyrus | 43 | **6939** | L | -60 | -8 | 12 | 11.20 | <0.001 |
| Superior temporal gyrus | 41/22 |  |  | -58 | -18 | 8 | 9.99 |  |
|  |  |  |  | -60 | -6 | 2 | 9.74 |  |
| Middle temporal gyrus | 21 |  |  | -58 | -20 | -2 | 9.50 |  |
|  |  |  |  | -54 | -20 | -4 | 9.27 |  |
| Thalamus | n/a |  |  | -14 | -20 | 6 | 8.01 |  |
| **Decreases** | | | | | | |  | |
| Parahippocampal gyrus | 37 | **293** | L | -32 | -40 | -8 | 9.82 | <0.001 |
| Precentral gyrus | 4 | **2701** | R | 28 | -18 | 38 | 7.47 | <0.001 |
| Precuneus | 31 |  |  | 22 | -42 | 30 | 6.81 |  |
|  |  |  |  | 22 | -50 | 32 | 6.23 |  |
| Superior temporal gyrus | 39 | **2105** | L | -30 | -56 | 32 | 7.13 | <0.001 |
| Cingulate gyrus | 24 |  | L | -18 | -16 | 40 | 6.90 |  |
|  |  |  | L | -24 | -22 | 36 | 6.77 |  |
| Superior temporal gyrus | 39 |  | L | -46 | -70 | 32 | 6.47 |  |
| Anterior cingulate cortex | 24 | **910** | L | -6 | 26 | 4 | 6.10 | <0.001 |
| Caudate | n/a |  | R | 10 | 20 | 8 | 5.66 |  |

| **Preserved insight patients** | | **Cluster size**  (voxels) | **Side** | **MNI** | | | Voxel  *T* Value | Cluster FWE-corrected *p* |
| --- | --- | --- | --- | --- | --- | --- | --- | --- |
| 1. **Self** | **BA** |  |  | **X** | **Y** | **Z** |  |  |
| **Increases** | | | | | | | | |
| Superior temporal gyrus | 41 | **1629** | L | -44 | -34 | 12 | 7.88 | <0.001 |
|  | 22 |  |  | -62 | -28 | 6 | 7.72 |  |
|  |  |  |  | -64 | -12 | 2 | 7.71 |  |
| Inferior frontal gyrus | 47 | **560** | R | 44 | 16 | -2 | 7.14 | 0.001 |
| Precentral gyrus | 6 |  |  | 62 | -2 | 10 | 5.51 |  |
| Postcentral gyrus | 43 |  |  | 66 | -16 | 16 | 4.19 |  |
| **Decreases** | | | | | | |  | |
| Caudate |  | **803** | R | 26 | -44 | 8 | 10.40 | <0.001 |
|  |  |  |  | 18 | -32 | 20 | 7.71 |  |
| Parahippocampal gyrus | 19 | **1135** | L | -28 | -46 | -2 | 9.65 | <0.001 |
|  | 30 |  |  | -20 | -40 | 6 | 8.50 |  |
| Caudate | n/a |  |  | -18 | -30 | 22 | 5.03 |  |
| **2. Self-distorted** | | | | | | | | |
| **Increases** | | | | | | | | |
| Superior temporal gyrus | 22 | **1547** | L | -60 | 2 | 4 | 9.78 | <0.001 |
|  |  |  |  | -48 | -18 | 8 |  |  |
| Postcentral gyrus | 40 |  |  | -52 | -24 | 18 |  |  |
| **Decreases** | | | | | | |  | |
| Parahippocampal gyrus | 30 | **291** | **L** | -26 | -52 | 8 | 5.56 | 0.022 |
| Posterior cingulate |  |  |  | -30 | -70 | 14 | 5.40 |  |
| Parahippocampal gyrus |  |  |  | -28 | -60 | 8 | 4.70 |  |
| **3. Other** | | | | | | | | |
| **Increases** | | | | | | | | |
| Superior temporal gyrus | 22 | **2113** | **R** | 56 | -14 | -2 | 9.17 | <0.001 |
|  |  |  |  | 64 | -36 | 6 | 7.43 |  |
|  |  |  |  | 54 | 14 | -2 | 6.61 |  |
| Thalamus | n/a | **803** | **R** | 12 | -18 | 2 | 8.75 | <0.001 |
|  |  |  |  | 14 | -8 | -2 | 6.02 |  |
|  |  |  | **L** | -4 | -28 | -8 | 5.60 |  |
| Superior temporal gyrus | 42 | **2332** | **L** | -66 | -24 | 6 | 8.07 | <0.001 |
|  | 22 |  |  | -54 | 10 | 0 | 7.88 |  |
|  | 41 |  |  | -54 | -28 | 12 | 6.94 |  |
| **Decreases** | | | | | | | | |
| Precuneus | 31 | **1996** | **L** | -10 | -62 | 24 | 12.78 | <0.001 |
| Caudate | n/a |  |  | -22 | -36 | 16 | 9.79 |  |
| Parahippocampal gyrus | 30 |  |  | -24 | -48 | 8 | 7.90 |  |
| Cingulate gyrus | 31 | **1310** | **R** | 30 | -42 | 14 | 8.49 | <0.001 |
|  |  |  |  | 26 | -42 | 24 | 7.95 |  |
| Precuneus | 31 |  |  | 12 | -60 | 28 | 7.88 |  |
| **4. Other-distorted** | | | | | | | | |
| **Increases** | | | | | | | | |
| Transverse temporal gyrus | 41 | **713** | **L** | -54 | -22 | 12 | 5.70 | 0.001 |
| Postcentral gyrus | 40 |  |  | -64 | -22 | 14 | 5.30 |  |
| Superior temporal gyrus | 22 |  |  | -60 | -38 | 16 | 4.74 |  |
| Precentral gyrus | 43 | **594** | **R** | 60 | -8 | 14 | 5.63 | 0.002 |
| Superior temporal gyrus | 22 |  |  | 60 | -12 | 6 | 5.11 |  |
|  |  |  |  | 58 | -2 | 2 | 5.00 |  |
| **Decreases** | | | | | | | | |
| Nil | | | | | | | | |

| **Poor insight patients** | | **Cluster size**  (voxels) | **Side** | **MNI** | | | Voxel  *T* Value | Cluster FWE-corrected *p* |
| --- | --- | --- | --- | --- | --- | --- | --- | --- |
| **1. Self** | **BA** |  |  | **X** | **Y** | **Z** |  |  |
| **Increases** | | | | | | | | |
| Superior temporal gyrus | 22 | **2057** | R | 62 | -28 | 4 | 13.26 | <0.001 |
|  |  |  |  | 54 | -48 | 8 | 9.87 |  |
| Postcentral gyrus | 40 |  |  | 58 | -24 | 18 | 9.61 |  |
| Inferior parietal lobule | 40 | **2189** | L | -52 | -32 | 26 | 11.40 | <0.001 |
|  |  |  |  | -64 | -34 | 28 | 9.96 |  |
| Postcentral gyrus |  |  |  | -52 | -30 | 18 | 9.32 |  |
| Inferior frontal gyrus | 47 | **284** | L | -32 | 30 | 0 | 6.26 | 0.027 |
|  |  |  |  | -30 | 24 | -8 | 5.90 |  |
| **Decreases** | | | | | | | | |
| Nil | | | | | | | | |
| **2. Self-distorted** | | | | | | | | |
| **Increases** | | | | | | | | |
| Cuneus | 19 | **856** | **L** | -28 | -86 | 22 | 10.89 | <0.001 |
|  |  |  |  | -22 | -92 | 24 | 7.67 |  |
| Superior parietal lobule | 7 |  |  | -22 | -62 | 42 | 7.35 |  |
| Superior temporal gyrus | 22 | **1797** | **R** | 54 | -30 | 4 | 9.58 | <0.001 |
| Inferior parietal lobule | 40 |  |  | 64 | -40 | 24 | 8.73 |  |
| Middle temporal gyrus | 21 |  |  | 60 | -6 | -4 | 7.70 |  |
| Superior temporal gyrus | 22 | **790** | **L** | -60 | -40 | 20 | 8.73 | <0.001 |
|  |  |  |  | -50 | -52 | 12 | 7.80 |  |
| Insula | 13 |  |  | -46 | -44 | 12 | 5.91 |  |
| Superior occipital gyrus | 19 | **708** | **R** | 32 | -92 | 18 | 7.90 | <0.001 |
|  |  |  |  | 38 | -76 | 20 | 7.05 |  |
| Precuneus | 19 |  |  | 30 | -76 | 30 | 6.28 |  |
| **Decreases** | | | | | | | | |
| Nil | | | | | | | | |
| **3. Other** | | | | | | | | |
| **Increases** | | | | | | | | |
| Superior temporal gyrus | 22 | **1440** | **R** | 68 | -38 | 10 | 8.73 | <0.001 |
|  |  |  |  | 52 | -32 | 2 | 8.28 |  |
|  |  |  |  | 60 | -14 | -6 | 6.27 |  |
| Superior temporal gyrus | 22/41 | **685** | **L** | -60 | -36 | 14 | 6.38 | <0.001 |
|  |  |  |  | -46 | -30 | 2 | 5.75 |  |
|  |  |  |  | -54 | -36 | 22 | 5.53 |  |
| Inferior frontal gyrus | 47/45 | **237** | **R** | 32 | 26 | -4 | 5.90 | 0.035 |
|  |  |  |  | 36 | 24 | 6 | 5.62 |  |
| **Decreases** | | | | | | | | |
| Caudate | n/a | **494** | **L** | -18 | 26 | -2 | 5.88 | 0.002 |
| Anterior cingulate cortex | 32 |  |  | 0 | 46 | -2 | 5.64 |  |
|  |  |  |  | 0 | 34 | 2 | 4.82 |  |
| **4. Other-distorted** | | | | | | | | |
| **Increases** | | | | | | | | |
| Superior temporal gyrus | 22 | **457** | **R** | 64 | -18 | 6 | 7.56 | <0.001 |
|  |  |  |  | 52 | -34 | 6 | 7.05 |  |
|  |  |  |  | 66 | -28 | 0 | 6.23 |  |
| Thalamus | n/a | **291** | **L** | -6 | -30 | -2 | 7.13 | 0.009 |
|  |  |  | **R** | 8 | -30 | -4 | 6.42 |  |
|  |  |  | **L** | -12 | -20 | 2 | 5.47 |  |
| Superior temporal gyrus | 22 | **462** | **L** | -56 | -46 | 14 | 6.03 | 0.001 |
|  |  |  |  | -60 | -38 | 6 | 5.92 |  |
|  | 41 |  |  | -46 | -32 | 4 | 5.82 |  |
| **Decreases** | | | | | | |  | |
| Cingulate gyrus | 31 | **223** | **R** | 16 | -46 | 26 | 10.58 | 0.024 |
|  |  |  |  | 28 | -16 | 22 | 6.12 |  |
|  |  |  |  | 30 | -28 | 30 | 5.48 |  |
| Precuneus |  | **244** | **L** | -22 | -58 | 30 | 5.93 | 0.018 |
|  |  |  |  | -26 | -50 | 32 | 5.05 |  |

*BA = Brodmann Area
