# Supplementary material for: Is it me? Verbal self-monitoring neural network and clinical insight in schizophrenia
Source: Psychiatry Res. 2015 Dec 30;234(3):328–35. doi: 10.1016/j.pscychresns.2015.10.007 (PMC4834462; doi:10.1016/j.pscychresns.2015.10.007)
Supplement: Supplementary file 2 — Supplementary material [file mmc2.pptx]

## Slide 1
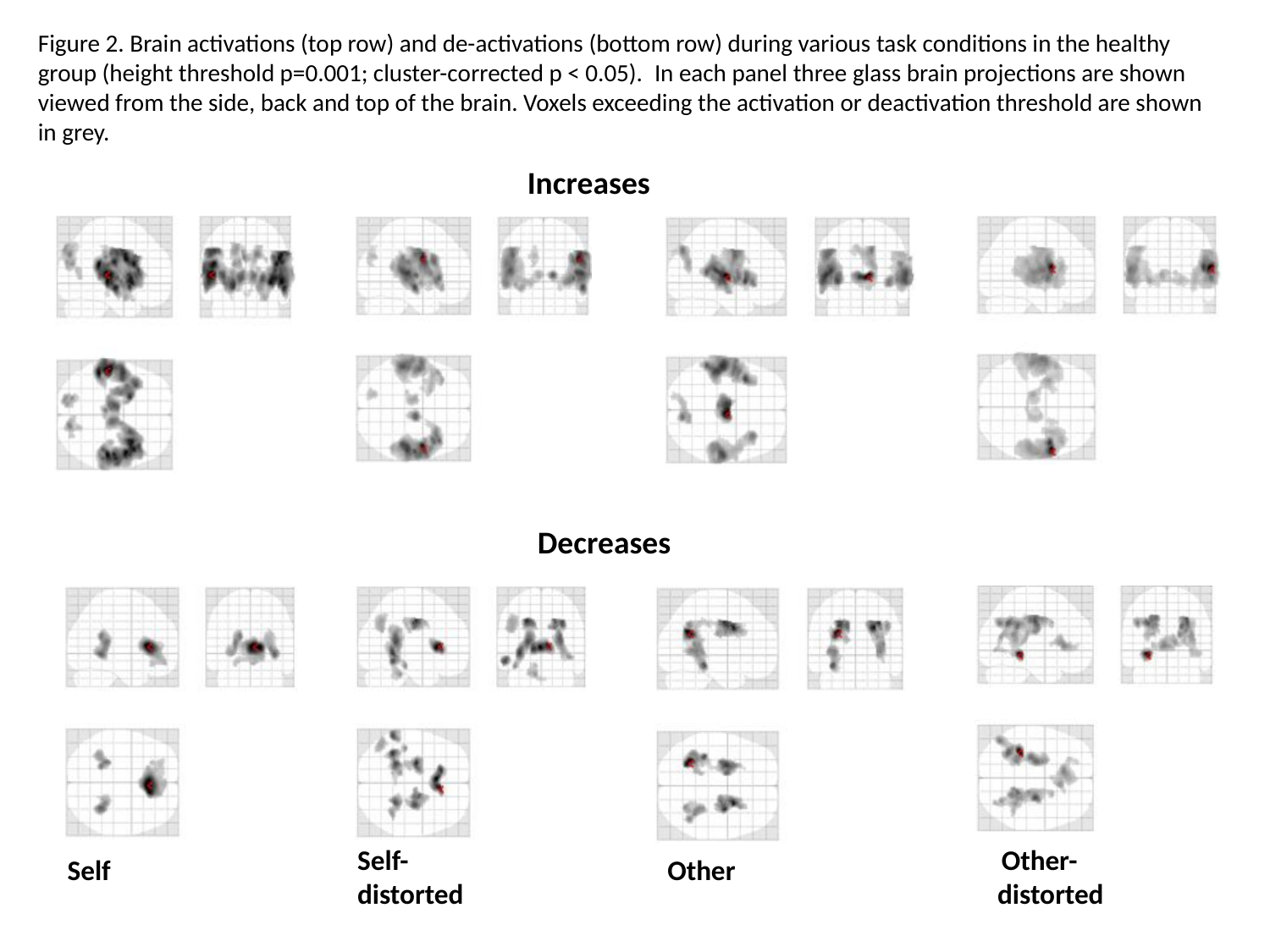

Figure 2. Brain activations (top row) and de-activations (bottom row) during various task conditions in the healthy group (height threshold p=0.001; cluster-corrected p < 0.05).  In each panel three glass brain projections are shown viewed from the side, back and top of the brain. Voxels exceeding the activation or deactivation threshold are shown in grey.
Increases
Decreases
Self-distorted
 Other-distorted
Self
Other
